# Pain assessment in emergency department as part of triage system has limited interobserver agreement

**DOI:** 10.1186/1757-7241-23-S1-A54

**Published:** 2015-07-16

**Authors:** Martin Hangaard, Brian Malling, Christian B Mogensen

**Affiliations:** 1 Department of Emergency Medicine, Hospital of Southern Jutland, Aabenraa, Denmark

## Background

In the Danish Emergency Process Triage (DEPT) pain is used as an independent contributor for triaging patients and is validated by the Numerical Pain Rating scale, NRS-11. Prior studies have assessed the congruence between nurse's assessment of their patients' pain intensity and the patients' own pain perception with mixed results. The aim of this study was to examine the correlation in pain score between nurse and patient and the inter-rater agreement between nurses when the pain score is used in a triage system in an Emergency Department.

## Method

The patients were asked to score themselves on the NRS scale and a final NRS score was then given by the nurses based on their objective findings. We asked two nurses to independently validate the same patients according to the DEPT triage instructions. The two nurses did not have access to each other's pain score of the patient. Data were analysed by Wilcoxon signed rank test and Kappa statistics.

## Results

A total of 326 independent patient-nurse pairs of observations were analysed. The nurses scored the patient significantly lower on the NRS scale than the patients did themselves (NRS median 3 versus 5, p < 0.0001). The overall inter-rater agreement between nurses and patient was moderate (Kappa 0.46; 95% CI 0.46-0.50) but lower for younger patients and patients with an orthopaedic pain complaint. In 163 patients, pain score was assessed by two nurses and the inter-rater agreement moderate (Kappa 0.54; 95% CI 0.49-0.61), lower for younger patients and female patients.

## Conclusion

Nurses score patient pain lower than the patients on the NRS scale, especially young and orthopaedic patients. The inter-rater agreement between the nurses was also moderate and lower for young and female patients. These findings weaken the usage of pain in triage systems.

